# Sharing longitudinal, non-biological birth cohort data: a cross-sectional analysis of parent consent preferences

**DOI:** 10.1186/s12911-018-0683-x

**Published:** 2018-11-12

**Authors:** Kiran Pohar Manhas, Shawn X. Dodd, Stacey Page, Nicole Letourneau, Carol E. Adair, Xinjie Cui, Suzanne C. Tough

**Affiliations:** 1 Community Health Sciences, University of Calgary, Calgary, Canada; 2 PolicyWise for Children & Families, Calgary, Canada; 3 University of Alberta, Edmonton, Canada; 4 Conjoint Health Research Ethics Board, University of Calgary, Calgary, Canada; 5 University of Calgary, Calgary, Canada; 6 PolicyWise for Children & Families, Edmonton, AB Canada; 7 Pediatrics & Community Health Sciences, University of Calgary, Calgary, Canada; 8 Alberta Health Services, Calgary, Canada

**Keywords:** Consent, Data sharing, Non-biological data, Parent, Pediatric, Data repository

## Abstract

**Background:**

Mandates abound to share publicly-funded research data for reuse, while data platforms continue to emerge to facilitate such reuse. Birth cohorts (BC) involve longitudinal designs, significant sample sizes and rich and deep datasets. Data sharing benefits include more analyses, greater research complexity, increased opportunities for collaboration, amplification of public contributions, and reduced respondent burdens. Sharing BC data involves significant challenges including consent, privacy, access policies, communication, and vulnerability of the child. Research on these issues is available for biological data, but these findings may not extend to BC data. We lack consensus on how best to approach these challenges in consent, privacy, communication and autonomy when sharing BC data. We require more stakeholder engagement to understand perspectives and generate consensus.

**Methods:**

Parents participating in longitudinal birth cohorts completed a web-based survey investigating consent preferences for sharing their, and their child’s, non-biological research data. Results from a previous qualitative inquiry informed survey development, and cognitive interviewing methods (*n* = 9) were used to improve the question quality and comprehension. Recruitment was via personalized email, with email and phone reminders during the 14-day window for survey completion.

**Results:**

Three hundred and forty-six of 569 parents completed the survey in September 2014 (60.8%). Participants preferred consent processes for data sharing in future independent research that were less-active (i.e. no consent or opt-out). Parents’ consent preferences are associated with their communication preferences. Twenty percent (20.2%) of parents generally agreed that their child should provide consent to continue participating in research at age 12, while 25.6% felt decision-making on sharing non-biological research data should begin at age 18.

**Conclusions:**

These finding reflect the parenting population’s preference for less project-specific permission when research data is non-biological and de-identified and when governance practices are highly detailed and rigourous. Parents recognize that children should become involved in consent for secondary data use, but there is variability regarding when and how involvement occurs. These findings emphasize governance processes and participant notification rather than project-specific consent for secondary use of de-identified, non-biological data. Ultimately, parents prefer general consent processes for sharing de-identified, non-biological research data with ultimate involvement of the child.

**Electronic supplementary material:**

The online version of this article (10.1186/s12911-018-0683-x) contains supplementary material, which is available to authorized users.

## Background

Data platforms, such as biobanks and secondary data repositories, are proliferating and facilitating innovation via large-scale data mobilization. Internationally, the funders and custodians of public research encourage or mandate, the sharing and re-use of research data, especially via data platforms [1–6]. The benefits of secondary research data use include (a) further analyses, replications, and verifications [1]; (b) increased diversity, novelty and complexity of research opportunities [5]; (c) decreased risk of “failure to discover” [7]; (d) more intra- and inter- disciplinary collaborations; (e) cost savings benefiting the public, funders, researchers and trainees [1]; (f) timely information for policy and practice; and, (g) maximized participant contributions and conservation of research resources. There are challenges to both privacy and consent when data are stored for such secondary use.

Privacy and consent are related: while privacy represents a right “to be let alone,” [8], consent transforms what may be a privacy violation into a permissible act [9]. In health research, informed consent allows individuals to exercise control over who may collect, view, use, disclose and store their personal information [10–12]. Only in exceptional circumstances can personal information be used without consent, with level of identifiability and research ethics board approval as key determinants of such exceptions [10–14]. Valid consent must be voluntary, informed, and provided by individuals with decision-making capacity [13, 15, 16]. Traditionally, in research, consent is operationalized through discussions between participants and researchers about the known research intent, including risks benefits and privacy considerations. In secondary data use, research intents cannot be fully, specifically known at the time of data collection.

Longitudinal cohort studies initiated prior to birth can be particularly rich as sources of secondary data [17]. Such studies involve large samples, multiple data collection points, and a wealth of information, including physical, emotional, developmental, social, and demographic data. Access to such data may permit strong study designs, greater statistical power and increased opportunities to examine diverse issues and relationships in the health, development, and well-being of children, mothers and families [17–20].

The benefits of secondary data use come with significant ethico-legal challenges including security and privacy protections, consent, governance, access and communication strategies [15, 21–30]. Pediatric populations add a dimension around consent and vulnerability [18–20, 31–36]. Very young children lack decision-making capacity, so parents or guardians act as surrogate decision-makers. As the child matures, decision-making capacity evolves and the applicability of parental consent becomes questionable. Risks of privacy breaches, discrimination or stigma noted for genomic studies introduce long-term risks when children are involved [18]. Longitudinal cohort data involving genetic information are, thus, flagged as requiring detailed consideration of ethical and legal safeguards for the child participants [18, 22, 37, 38]. However, biobank and epidemiologic data differ and standards originally developed for biological data may be overly restrictive, inappropriate, or may disregard unique concerns when applied to non-biological data [39]. For example, the risks of re-identification and familial implication can be better redressed with epidemiologic, compared to biological, data [39].

The appropriate consent process for secondary use is unclear. Because the intent of future research is unknown at the point of primary collection, project-specific consent is impossible. Cost and feasibility issues hinder re-contacting participants for each secondary use within large-scale data platforms. Consensus is lacking on (a) the most appropriate form of consent for biobanks and repositories; (b) if/how participants can withdraw their data from repositories; (c) how best to communicate between repositories, researchers and participants about re-use and findings; (d) how child age or experience influences child assent and dissent; (e) how consent is influenced by parent-child disagreement; and (f) the feasibility issues for pediatric biobanks and repositories [15, 16, 36, 40, 41]. This lack of consensus produces logistical, ethical and legal obstacles for data repositories.

Currently, Canadian research ethics policy (TCPS2) permits secondary use of research data to proceed without consent, if data are de-identified and research ethics approval is gained (Article 5.5B, TCPS2) [13]. This balances the risks to individuals against the benefits to society from data use and knowledge advancement. Secondary use of identifiable, or potentially identifiable, data hinges on consent. Waiver of consent is possible, if several, specific criteria are met including the importance of the research question outweighing the potential harms of the individuals and the infeasibility of garnering consent (Article 5.5A, TCPS2) [13]. For children, the TCPS2 has moved from age-based restrictions on consent to capacity, so that children under the age of majority may consent when deemed capable [13]. Capacity is generally considered on a case-by-case basis, which introduces feasibility and cost hurdles for secondary data repositories and researchers.

**Fig. 1:**
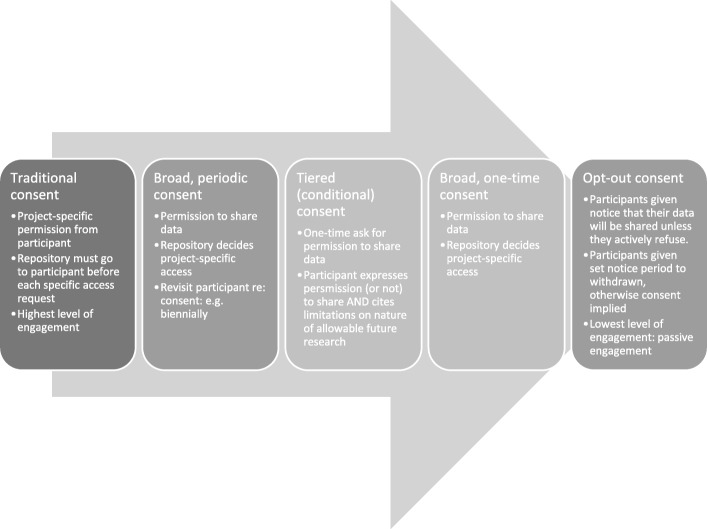
Definitions of the five consent models examined in this survey (from highest to lowest level of engagement)

Consent processes can vary based on level of engagement. The five most commonly-considered consent processes span high to low levels of engagement: (1) the traditional consent model; (2) broad, periodic consent model; (3) broad, one-time consent model; (4) tiered (or conditional) consent model; and, (5) opt-out consent model (Fig. 1). It should be noted that opt-out consent is a model discussed in the bioethics literature but is not a consent mode recognized by law, or the TCPS2, in Canada. The traditional consent model is project-specific and involves high-level engagement; the three broad- or tiered- consent models are focused on enabling secondary use broadly, while the final, opt-out consent model utilizes passive engagement with consent implied if permission is not actively withdrawn.

TCPS2 advocates engagement with relevant populations to seek input on ethical issues and appropriate privacy protection [13]. Parents are critical stakeholders, as they are the gatekeepers to data. Parent perspectives have been solicited about biobank participation for their children [42–45] and for themselves [43, 46]. Parent concerns impacting agreement to repository participation include lack of information about future uses; risks of stigma; privacy or consent issues; researcher credibility questions; and inability to be re-contacted for results [43, 44, 46]. Ethnicity appears to impact biobank participation in the US, with minorities more reticent than Caucasians [46, 47].

Few studies have specifically examined parent consent preferences. In a structured-interview-based study, 84 parents in hospital-based pediatric clinics were asked their opinion on the layout of a draft biorepository opt-out consent form [42]. Only one parent explicitly stated he preferred opt-in consent and eight parents wished for more information [42]. Because parents were not asked to compare or even consider other consent processes, this perceived acceptability of opt-out consent for biobanking is incomplete. In another study, 166 parents of children in pediatric wards were surveyed about consent preferences for hypothetical observational research [48]. Fifty-two percent chose an opt-in consent approach, 33% selected opt-out, and about 15% felt that consent was unnecessary [48]. The generalizability of consent preferences for observational research compared to consent for secondary data use is unclear as the latter is characterized by greater diversity and uncertainty.

Another recent study compared parent (*n* = 113) and adult (*n* = 196) participants from six genomic studies on data sharing consent [45]. While adults and parents are not mutually exclusive, this study investigated the perspective of parents (mostly mothers) versus that of adults who do not have children for whom surrogate decision-making is required. Parents and adult participants were randomly assigned to one of three experimental consent forms (traditional, broad and tiered), and asked whether they would release their child’s data publicly and openly, with restrictions and access processes, or not release at all [45]. This study looked more at parents versus adults and the access level permitted for sharing, rather than preference amongst consent processes. Parents were found to be significantly more restrictive in data release decisions than adults because of autonomy and control preferences, not understanding or perceived benefits; parents also always selected the more restrictive data sharing option when such option was available to them for sharing their child’s data [45]. No studies were found of parent’s willingness to enrol themselves or their children in a data repository or their preferences for future consent for use of their or their child’s data, or on preferred consent models for this circumstance.

A few studies have solicited child and adolescent preferences directly. A study of five focus groups with adolescents yielded their concerns with biobanking as including parental involvement, growing autonomy, information needs, benefits and burdens [49]. Adolescents trusted their parents, but requested engagement in decision-making as they aged [49]. A qualitative study of 21 seven-year-olds elicited their general experience participating in a longitudinal birth cohort [50]. Children involved in longitudinal research enjoyed active participation (e.g. involving moving, running, or playing with a computer), but disapproved of venipunctures, some to the point of refusal [70]. Minors generally felt that researchers should re-contact children upon reaching majority for consent, although this was not considered mandatory with best-efforts requested [49].

A recent qualitative study with 55 adolescents aged 17–19 years who were involved in a birth cohort, from before their birth, examined views around data sharing via data linkage of cohort data [51]. This study found that although different consent processes were explained, participants viewed consent generally as ‘opt-in’ consent where project-specific permission was requested [51]. Participants raising similar concerns (e.g. social sensitivity of research question, questionable effectiveness of anonymization, ownership of personal information) came to different conclusions on whether consent was needed [51]. Adolescent views changed when presented with alternative scenarios and were somewhat inconsistent [51]. The authors of this study questioned “… the validity of ‘informed consent’ as a cornerstone of good governance, and the extent to which potential research participants understand different types of consent and what they are consenting, or not consenting, to” [51]. This dearth of child and adolescent perspectives on consent for data sharing whether biological or non-biological data calls for further empirical understanding of stakeholder perspectives on consent preferences when sharing non-biological research data of parents and children in longitudinal research.

To fill these gaps, this study was conducted to address “what are parents preferences amongst alternatives in the secondary use of their and their child’s non-biological, de-identified birth cohort data?”

## Methods

A cross-sectional, online survey was used to assess consent preferences of parents enrolled in two Alberta birth cohorts for the secondary use of adult and child non-biological, de-identified cohort data via a non-biological data repository. This survey represents the final stage of a mixed-methods study examining parental views on privacy, consent and governance in secondary data use; the qualitative findings that preceded and informed survey development are published elsewhere [52–54].

### Study sample

Participants were recruited from two provincial longitudinal birth cohorts, All Our Families (AOF) [15] and Alberta Pregnancy Outcomes and Nutrition (APrON) [16]. These two cohorts began in 2007 and are prospective, community-based birth cohorts, situated in Alberta, Canada. Together these cohorts include approximately 5200 mother-baby pairs and 1200 fathers. Detailed overviews of the cohorts’ methods and design, are described elsewhere [55, 56].

Of the participants who consented to re-contact for future research, cohort research assistants contacted a randomly-generated list of participants to obtain permission to share contact information for this survey’s recruitment. Those who agreed received a personalized, email invitation to complete the survey. Pre-notification of the survey and its purpose was provided. Participants from earlier phases of the study were excluded from the online survey.

### Online survey development

The online survey was administered via the University of Alberta’s Electronic Patient Reported Outcomes (ePRO) platform. Survey design was based on previous qualitative findings, a literature review of participant and public and parent perspectives on data sharing, and pre-testing using cognitive interviewing (CI) [53, 54].

The CI pre-test involved one-on-one interviews (*n* = 9) [57]. CI recruitment followed the qualitative study [53, 54]. During CI interviews, participants completed the draft survey on ePRO (or on paper as needed), while receiving verbal probes from the CI interviewer [57]. Questions explored respondents’ understanding and preferences around survey layout, questions, and choices [57]. Specifically, item content, construction, comprehension, confidence judgment, and possible participant reactions were addressed [32, 57]. Concurrent analyses of CI findings involved text-based and thematic analysis. The survey was revised after every other CI; revisions were then discussed in subsequent CIs.

The final survey contained five sections: (a) parents’ motivations and reservations surrounding research, data and data repositories; (b) protective and organizational approaches for data repositories; (c) consent preferences; (d) sensitivity of pediatric data and secondary research using this data; and (e) preferences for modes of communication between data repository administrators and parent participants. The survey used fixed-choice response options and Likert scales. Previous qualitative work suggested that the survey topic was not commonly discussed in this population [53, 54]. Background information, particularly on repository governance, was thus provided to promote shared understanding (summarized in Additional file 1).

In this survey, child involvement in research consent discussions and in secondary data use was innovatively presented as three phases: tell, talk and decide. The ‘tell’ phase encompassed when parents informed their child of their participation. The ‘talk’ phase considered when parents involved their child in two-way discussions, including child’s expression of opinions and preferences. In both the ‘tell’ and ‘talk’ phases, parents decided the child’s participation. The final ‘decide’ phase encompassed when parents allowed their child to decide their own participation. When a parent respondent agreed with the utility of any phase, they were to indicate the child’s age to start that phase.

The initial, personalized email invitation requested survey completion within 2 weeks. Reminders were emailed on days 3 and 11 [58]. The AOB participants received a follow-up call on day 7 to ensure receipt of the invitation and to answer questions [58]. Upon survey completion, participants could submit their e-mail for a lottery (prize: iPod Touch); this information was segregated from survey data. The study was approved by the Conjoint Health Research Ethics Board at the University of Calgary.

### Data analysis

The data were summarized using frequency distributions. Chi-squares and Fisher’s exact tests were used to test for associations between participant motivations and reservations towards data sharing, communication preferences and consent preferences. Missing responses varied by question. Reported percentages are item-specific (denominator varies).

The relationship between desired level of engagement and preferred consent model was examined. Consent models were collapsed into three groups: traditional consent, broad consents, and opt-out consent. The broad-consents category included broad, one-time consent; broad, periodic consent; and tiered consent. When comparing parent’s level of agreement to involving their child in decision-making, the five-point scale was collapsed. The response options “strongly agree” and “agree” were collapsed into “agree”; “strongly disagree” and “disagree” were collapsed into “disagree.” STATA for Mac version 14.1 was used and a significance level of *p* < 0.05 set.

## Results

Three hundred and forty-six participants completed the survey from 569 personalized invitations sent in September 2014 (response rate = 60.8%). This included 106 AOB participants, 190 APrON participants, and 50 participants who were members of both cohorts (Table 1). Amongst the survey respondents, 86.7% were Canadian; 98.0% were female; 69.1% were over the age of 35. Most (96.2%) had some post-secondary education.

**Table 1:** Participant demographics

Characteristic	n (%)
Longitudinal Birth Cohort	
All Our Babies	105 (30.3)
Alberta Pregnancy Outcomes and Nutrition	188 (54.3)
Both	50 (14.5)
Missing	3 (0.8)
Sex	
Male	5 (1.4)
Female	339 (98.0)
Missing	2 (0.5)
Age (years of age)	
< 35	110 (31.8)
> 35	208 (69.1)
Missing	28 (8.1)
Education	
High School (Completed)	9 (2.6)
Business, Trade, Technical School (Incomplete or completed)	51 (14.7)
Bachelor’s Degree (Incomplete or completed)	190 (54.9)
Graduate School (Incomplete or completed)	92 (26.6)
Missing	4 (1.2)
Country of Origin	
Canada	300 (86.7)
Other	34 (9.8)
Missing	12 (3.4)

### Preferred engagement for consent process

This survey queried participants on the five most commonly considered consent processes: traditional consent; broad, periodic consent; broad, one-time consent; tiered consent; and, opt-out consent (Fig. 1). All questions around consent centred on the research best practice of using de-identified data, which cannot completely anonymize a dataset especially when opportunities exist to combine data from multiple sources or perspectives.

About 55.8% of respondents felt that their consent should be obtained before their de-identified, non-biological data is provided to a data repository for future, secondary use. These respondents were then asked their preferred process for contributing their data to a repository for secondary use (Table 2). The near-majority preferred the least-engaging opt-out consent process (47.2%), while the next most favourable process was the highly-engaging traditional consent (21.9%).

**Table 2:** Participant consent preferences for secondary use

	Preferred consent model if asked to share data from their original cohort n(%)	Preferred consent model if participating in a new study n(%)
Traditional, Opt-In Consent (Participant asked each time dataset shared)	75 (21.9)	78 (22.8)
Broad, One-Time Consent (Participant is asked permission one-time at beginning for all future uses)	33 (9.6)	35 (10.2)
Broad, Periodic Consent (Participant is asked every two years for permission to continue sharing data)	33 (9.6)	25 (7.3)
Tiered (or Conditional) Consent (Participant identifies allowable uses of their data when granting permission)	40 (11.7)	41 (12.0)
Opt-Out Consent (Participant must declare withdrawal within 3 months or permission is assumed)	162 (47.2)	163 (47.7)

Respondents were asked to rank each consent process on six domains including respectfulness, cost, convenience, informative-ness, feasibility, and control before selecting their preference. Previous qualitative findings in this population suggested that such focussed consideration might better-inform consent preferences (Additional file 2), [53, 54]. Most respondents rated traditional opt-in consent as the most expensive (89.0%), most informative (75.8%), and most affording of control to participants (79.3%). Opt-out consent was ranked lowest on cost (1.6%), informative-ness (2.2%), respectfulness (2.6%), and, tied with broad one-time consent, on control (3.2%). Considering respect and convenience, respondent perceptions were divided. The three processes most often cited as respectful were traditional opt-in (54.5%), tiered (18.3%), and broad-periodic consent (14.7%). The three processes most often cited as convenient were broad, one-time (40.4%), tiered (23.4%) and opt-out consent (23.4%).

Respondents indicated that their preferred means to communicate consent was by email (71.0%), with a password-protected online account (16.1%) being the second-most preferred option. Telephone was the least preferred communication mode (5.7%).

Respondents considered consent processes for a hypothetical new research study that anticipated the to-be-collected data would be shared upon study completion (Table 2). Again, respondents were most supportive of the opt-out consent model (47.7%) followed by the traditional consent model (22.8%).

### Future communication preferences vs. consent preferences

Respondents were asked what information would most interest them from a research data repository that housed and shared their or their child’s data. The majority of respondents were interested in information pertaining to specific projects that used their dataset (86.7%) and general findings from their dataset (87.9%). Respondents were less interested in general findings arising from the repository’s full complement of datasets (35.5%) and information about administrative changes (40.2%). Respondents indicated preferences with the frequency and mode of communication with repositories. Respondents most frequently wanted to hear from the repository once a year (39.2%) through a personalized email (50.1%) or a general newsletter (39.4%).

Statistically significant associations were detected between consent preference and communication frequency and information type (Table 3). Respondents who preferred more engaging models of consent (traditional or broad consent models), were more interested in receiving information pertaining to projects that arose from their dataset (*p* = 0.004), general findings from the repository (*p* = 0.033) and changes to the repository (*p* = 0.035). Respondents preferring engaging consent also wished for more frequent communication (*p* = 0.003).

**Table 3:** Relationships between consent preferences, communication frequency and information type

	Preferred consent model if asked to share their data from their original cohort. Consent should be reviewed…			
	Each time dataset is shared n(row/column%)	Periodically n(row/column%)	Never n(row/column%)	p-value
Are you interested in receiving information about projects that arise from the study you participated in?				
Yes	73 (24.58/97.33)	92 (30.98/86.79)	132 (44.44/81.48)	0.004
No	2 (4.35/2.67)	14 (30.43/13.21)	30 (65.22/18.52)	
Are you interested in receiving information about the general findings from the dataset you contributed to?				
Yes	68 (22.59/90.67)	92 (30.56/86.79)	141 (46.84/87.04)	0.684
No	7 (16.67/9.33)	14 (33.33/13.21)	21 (50.00/12.96)	
Are you interested in receiving information about the general findings from all the data at the data repository?				
Yes	36 (29.75/48.00)	34 (28.10/32.08)	51 (42.15/31.48)	0.033
No	39 (17.57/52.00)	72 (32.43/67.92)	111 (50.00/68.52)	
Are you interested in receiving information about any changes made to the data repository?				
Yes	35 (25.55/46.67)	49 (35.77/46.23)	53 (38.69/32.72)	0.035
No	40 (19.42/53.33)	57 (27.67/53.77)	109 (52.91/67.28)	
Based on the information you would like to receive, how often would you most like to hear from the data repository?				
Anytime new information arises	30 (34.09/41.10)	17 (19.32/16.04)	41 (46.59/26.11)	0.003
Every 6 months	23 (23.71/31.51)	33 (34.02/31.13)	41 (42.27/26.11)	
Once a year	18 (13.43/24.66)	52 (38.81/49.06)	64 (47.76/40.76)	
Every other year	2 (11.76/2.74)	4 (23.53/3.77)	11 (64.71/7.01)	
Please indicate your most preferred method of contact.				
A personalized email	41 (23.98/55.41)	60 (35.09/57.14)	70 (40.94/43.75)	0.060
A general newsletter	21 (15.67/28.38)	39 (29.10/37.14)	74 (55.22/46.25)	
A secure, regularly-updated website only open to participants	9 (33.33/12.16)	5 (18.52/4.76)	13 (48.15/8.12)	
A secure, regularly-updated website open to the public	3 (42.86/4.05)	1 (14.29/0.95)	3 (42.86/1.88)	

### Consent preferences for Child’s data

**Fig. 2:**
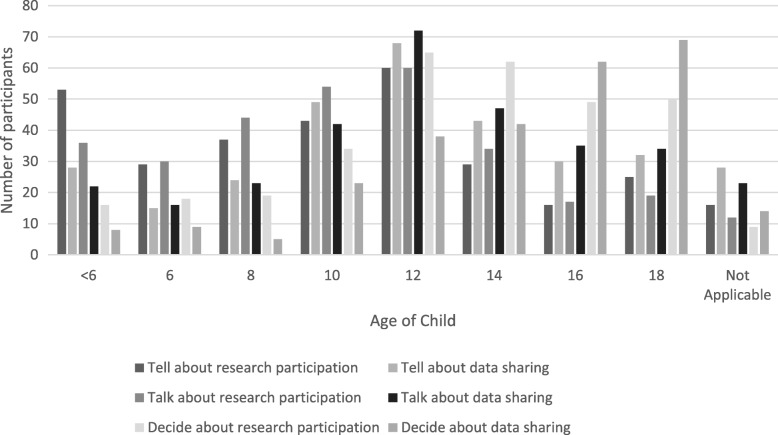
Parent views on appropriate child ages to “tell”, “talk” and “decide” about research participation and data sharing

Parents generally agreed (79.0%) that, at some point, they would tell their child of their involvement in a longitudinal research project (Table 4). This sentiment was shared in both the ‘talk’ (74.6%) and ‘decide’ (89.2%) phases. Despite this widespread agreement to involve children, the age at which to begin each phase varied substantially. For the ‘talk’ and ‘tell’ phases, parent’s felt most frequently comfortable involving children at 12 years of age (19.5%) with a median age of 10 (Fig. 2). Twelve years of age represented the mode (20.2%) and median age for parents feeling comfortable to let children act as primary decision-maker.

**Table 4:** Parent preferences on child involvement in decision-making for longitudinal research participation

	Level of agreement with child involvement in RESEARCH STUDIES			Level of agreement with child involvement in DATA SHARING		
	Disagree	Neutral	Agree	Disagree	Neutral	Agree
Tell - My child will be informed that they are part of a research study/their non-biological, de-identified data is being shared. As their parent. I decide the availability of their participation.	36 (10.5)	39 (11.3)	271 (79.0)	26 (7.5)	36 (10.4)	283 (82.1)
Talk - My child will be involved in discussions about their participation in the research study/sharing their non-biological, de-identified data. They can express their opinion and preferences. As their parent, I will decide their participation.	39 (11.4)	51 (14.8)	255 (74.6)	28 (8.2)	37 (10.8)	277 (81.0)
Decide - My child will decide whether or not, and how, they participate in the research study/share their non-biological, de-identified data.	21 (6.1)	19 (5.5)	305 (89.2)	75 (21.8)	46 (13.3)	224 (64.9)

Parents provided input on involving children in decision-making for secondary data use. Wide support by respondents for child involvement was expressed for the ‘talk’ and ‘tell’ phases (82.0% and 81.0%, respectively) when discussing data sharing (Table 4). Although the majority of respondents were supportive of the ‘decide’ phase (64.9%), there was a decrease in support for secondary use compared to the ‘decide’ phase for research participation.

When asked the appropriate age to begin child involvement in data sharing decisions, respondents expressed similar preferences to those for research participation. Twelve years of age represented both the mode (21.5% and 22.9%) and the median age for telling and talking (respectively) with their child about data sharing (Fig. 2). The age increased for when respondents felt comfortable allowing their child to act as primary decision-maker. Most respondents preferred 18 years (25.6%), whereas the median age was 16 years old. Finally, the majority of respondents (63.8%) favoured the repository informing parents of the child’s decision regarding data sharing.

## Discussion

This survey reveals that the majority of parents prefer less-engaging consent processes when discussing secondary data use of de-identified, cohort data for themselves and their children. Parents’ consent preferences are associated with their communication preferences. Parents also recognize that children should become gradually involved in consent decision-making around research and secondary data use participation. However, parents vary greatly on when and how that involvement should arise.

When compared to local and provincial data sources, the cohort participants are generally representative of the pregnant and parenting population in Calgary and Alberta, [59]. This study provides evidence of parent perspectives on data sharing, with certain limitations. First, this study population may be more supportive of research than the general population which may overestimate the support of the general population towards data sharing. Secondly, topic complexity may have influenced understanding and preferences. Detailed background information was provided to participants for each section of the survey, which may have altered participants’ perspectives.

When longitudinal birth cohort data are first collected, parental permission is sought for the secondary use of both parent and child data. In this study, it appears that parent-respondents are interested in fairly low levels of engagement in being asked for permission to share de-identified cohort data. About 44% of respondents did not feel consent was needed before a dataset including their and their child’s de-identified data were shared, consistent with the TCPS2 standard. Those participants were therefore excluded from the questions on consent preferences. Thereafter, a modest majority of parents preferred that they be asked for their consent generally. Nearly half of those consent-preferring parents specifically chose the least-engaging opt-out consent process. This intimates that the majority of respondents overall want no to low levels of engagement in consent for sharing their de-identified non-biological data.

In selections of the most preferred consent process, the remaining 55.8% of respondents were divided: about half preferred the least engaging opt-out model, which they also considered the least expensive, least informative, least controlling, and least respectful. Only 23.4% of respondents considered opt-out consent to be most convenient of the five consents presented. Participants were not different, from a practical standpoint, when considering consent for first-time primary research participation versus secondary data use. When the three broad consents were collapsed, respondent preference did not differ between broad consents and traditional, project-specific consent.

There did not appear to be any consensus in the ranking of consent model preferences; but there was a clear trend that requests for consent are not the highest priority for this parent population. Parents seem to recognize the implications of not asking for consent or going with a low-engagement model: less information, less control, less cost, and less respect. These findings support previous research that identified parent preference of opt-out consent in biobanking [42]. This survey moves beyond another to note that parents preferred opt-out (or no consent) over other processes, rather than simply not voicing an objection, [48]. This coincides with a recent systematic review that suggested that no consensus exists on the most appropriate consent for biobanks [16].

Parents were asked specifically about sharing de-identified, non-biological cohort data, which distinguishes the findings greatly from other parent engagement research around consent and biobanking [42–47]. It may be somewhat inappropriate and unduly restrictive to consider consent preferences for biological data as synonymous for those with non-biological data. There may be differences, particularly more comfort, in sharing de-identified non-biological data versus de-identified biological data: either because there is greater confidence in the ability to truly divest identifiable information from non-biological data versus biological data, or because there is greater trust in researchers and research involved with this parent population. In the latter case, survey respondents came from a long-term cohort study and previously consented to consider additional research. They may be more supportive of, and trusting in, research than other parent populations. The survey also elaborated the governance mechanisms involved in regulating data access, and participants provided input on such mechanisms. It is unclear how influential this discussion and questioning were on consent preferences. Our findings on governance (discussed elsewhere [60]), suggest that governance elements were more important to parents than privacy issues. These elements include the criteria and processes required for gaining access to data; the monitoring of data access; and, the bodies involved in governance of secondary use. Further research is required to determine the role, if any, of governance features on participants’ consent preferences for secondary data use.

Communication preferences largely mirrored consent preferences. Respondents most wanted limited engagement: yearly contact through one-way communications such as email or newsletter. There were corresponding increases in communication specificity and frequency desired among respondents preferring more engaging consent processes. This relationship makes sense and is consistent with a perspective that less interaction is necessary when long-term research participants share non-biological, de-identified data. Less desired interaction signalled greater trust in researchers, research and repositories. These findings may not be generalizable to all secondary use instances, but only to those with structured governance and data access processes including scientific review, qualification criteria, research ethics board approval, formal approval by the governing privacy authority, up-to-date information technology, a non-profit and research-focused agenda, and, sharing de-identified, non-biological datasets.

The majority of parent respondents supported gradual inclusion of children in decision-making for research and secondary data use. Amongst great variability, 12 years seemed the age at which most parents would inform children of, and begin discussions around, their information being used in research and secondary use. Decision-making for specific research would transition to children from parents around 12 years old. Decision-making for secondary data use, which has greater uncertainty on the specifics, logistics and implications of information use, fell more in line with legal definitions of majority at 18 years which contrasts current Canadian standards that demarcate capacity not age as the threshold for research decision-making. Parent views coincide with the scant literature on adolescent perspectives on data sharing [49–51], in that there is great variability but a general favouring of re-consent at the age of majority. Previous research with adolescents favoured a renewed focus on governance rather than consent when approaching secondary use of longitudinal cohort data [51].

## Conclusions

Both parents and youth involved in cohorts recognize the value of their data for secondary use and knowledge advancement. The consent conversation may not be crucial to garner access to that valuable data resource. Flexible, trustworthy approaches to the governance of data sharing are necessary to ensure long-term access to these data assets and safeguard public trust in research. Ensuring data storage is highly secure and up-to-date is crucial, as is confirming data access is restricted to approved researchers and entities and monitoring access and its outputs. Trust in institutions seems to move the conversation and responsibility away from parents and child participants to the researchers and repositories for de-identified, non-biological data [40]. This movement may be appropriate because parents and children lack the experience and capacity to assess and monitor the appropriateness and security of all future secondary data users.

## Additional files

Additional file 1: Overview of background material provided for each section of the survey. This table overviews the background information provided to participants in each section of the survey, given the findings 635 in the qualitative work that this population required some background 636 information to inform their preferences. (DOCX 17 kb)

Additional file 2: Parents’ perspectives on ranking consent models. This table reveals how parent respondents’ ranked each consent model on 639 features such as respectfulness, cost, convenience, and informativeness. (DOCX 16 kb)

## Abbreviations

AOF: All our families
APrON: Alberta pregnancy outcomes and nutrition
CI: Cognitive interviewing
ePRO: Electronic patient reported outcomes
