# Supplementary material for: Sharing longitudinal, non-biological birth cohort data: a cross-sectional analysis of parent consent preferences
Source: BMC Med Inform Decis Mak. 2018 Nov 12;18:97. doi: 10.1186/s12911-018-0683-x (PMC6233367; doi:10.1186/s12911-018-0683-x)
Supplement: Supplementary file 1 — Overview of background material provided for each section of the survey. This table overviews the background information provided to participants in each section of the survey, given the findings 635 in the qualitative work that this population required some background 636 information to inform their preferences. (DOCX 17 kb) [file 12911_2018_683_MOESM1_ESM.docx]

**Additional File 1.** Overview of Background Material Provided for Each Section of the Survey

| Topic | Overview |
| --- | --- |
| **Research, Data & Repositories** | - Data collected during research can take many forms   - Measurements, answers on questionnaires, etc.   - May be highly personal/sensitive or less personal/sensitive - Primary research are those who originally collect data - Secondary researchers use data collected by another researcher to answer new research questions (re-use the data) - Many research funders require data to be re-used - Research data repository is a please data can be stored, listed and distributed to secondary researchers - Data stored and shared by data repositories are entered into datasets - A dataset is all the information from each participants entered into a single document or table - Datasets can be de-identified by removing some or all identifying information |
| **Protective & Organizational Approaches** | - A data repository is being planned for Alberta, Canada called the Child Data Centre of Alberta (CDCA) - CDCA is being set-up so that it will:   - follow applicable laws in Alberta and Canada (ie. data protection laws)   - be approved by the Information and Privacy Commissioner of Alberta   - follow the industry best practices by looking at what other similar data repositories are doing   - use the most up-to-date information technology (IT) in the storage, use and management of data   - approval of secondary researchers would include applications forms, qualification criteria and a committee-based decision   - shared data would be compiled into de-identified, non-biological datasets containing the information you and other participants provided during research |
| **Permission (or Consent)** | - Traditionally, researchers explain specific aims, methods and implications of a project before participants decide to participate - For repositories there are many unknowns regarding future secondary researchers and their projects   - Ie. how many secondary researchers, kind of projects proposed, etc. - This complicates the consent process |
| **Children’s Data & Secondary Research** | - CDCA will store and share data collected during childhood and possibly into adolescence - CDCA aims to be a long-term research resource and therefore data may be stored into participants adulthood - Concerned with how to recognize children as they mature and develop their own opinions and preferences |
| **Communication & Notification** | - Interested in understanding participants preferences on how they would like to be communicate with data repository regarding their data and its uses |

**Supplement 2**. Parents’ Perspectives on Ranking Consent Models

|  | **Traditional Opt-in** | **Broad, One-Time** | **Broad-Periodic** | **Tiered** | **Opt-Out** | **Missing** |
| --- | --- | --- | --- | --- | --- | --- |
| **Most Respectful** | 104 (54.5) | 19 (9.9) | 28 (14.7) | 35 (18.3) | 5 (2.6) | 155 |
| **Most Expensive** | 162 (89.0) | 5 (2.7) | 10 (5.5) | 2 (1.1) | 3 (1.6) | 164 |
| **Most Convenient** | 16 (8.5) | 76 (40.4) | 29 (15.4) | 44 (23.4) | 23 (23.4) | 158 |
| **Most Informed** | 141 (75.8) | 6 (3.2) | 15 (8.1) | 20 (10.8) | 4 (2.2) | 160 |
| **Most Realistic** | 20 (10.6) | 55 (29.3) | 52 (27.7) | 47 (25.0) | 14 (7.4) | 158 |
| **Most Control** | 149 (79.3) | 6 (3.2) | 12 (6.4) | 15 (8.0) | 6 (3.2) | 158 |
