# Supplementary material for: Sharing longitudinal, non-biological birth cohort data: a cross-sectional analysis of parent consent preferences
Source: BMC Med Inform Decis Mak. 2018 Nov 12;18:97. doi: 10.1186/s12911-018-0683-x (PMC6233367; doi:10.1186/s12911-018-0683-x)
Supplement: Supplementary file 2 — Parents’ perspectives on ranking consent models. This table reveals how parent respondents’ ranked each consent model on 639 features such as respectfulness, cost, convenience, and informativeness. (DOCX 16 kb) [file 12911_2018_683_MOESM2_ESM.docx]

**Additional File 2.** Parents’ Perspectives on Ranking Consent Models

|  | **Traditional Opt-in** | **Broad, One-Time** | **Broad-Periodic** | **Tiered** | **Opt-Out** | **Missing** |
| --- | --- | --- | --- | --- | --- | --- |
| **Most Respectful** | 104 (54.5) | 19 (9.9) | 28 (14.7) | 35 (18.3) | 5 (2.6) | 155 |
| **Most Expensive** | 162 (89.0) | 5 (2.7) | 10 (5.5) | 2 (1.1) | 3 (1.6) | 164 |
| **Most Convenient** | 16 (8.5) | 76 (40.4) | 29 (15.4) | 44 (23.4) | 23 (23.4) | 158 |
| **Most Informed** | 141 (75.8) | 6 (3.2) | 15 (8.1) | 20 (10.8) | 4 (2.2) | 160 |
| **Most Realistic** | 20 (10.6) | 55 (29.3) | 52 (27.7) | 47 (25.0) | 14 (7.4) | 158 |
| **Most Control** | 149 (79.3) | 6 (3.2) | 12 (6.4) | 15 (8.0) | 6 (3.2) | 158 |
